# Supplementary figures and images for: ProteoClade: A taxonomic toolkit for multi-species and metaproteomic analysis
Source: PLoS Comput Biol. 2020 Mar 9;16(3):e1007741. doi: 10.1371/journal.pcbi.1007741 (PMC7082058; doi:10.1371/journal.pcbi.1007741)

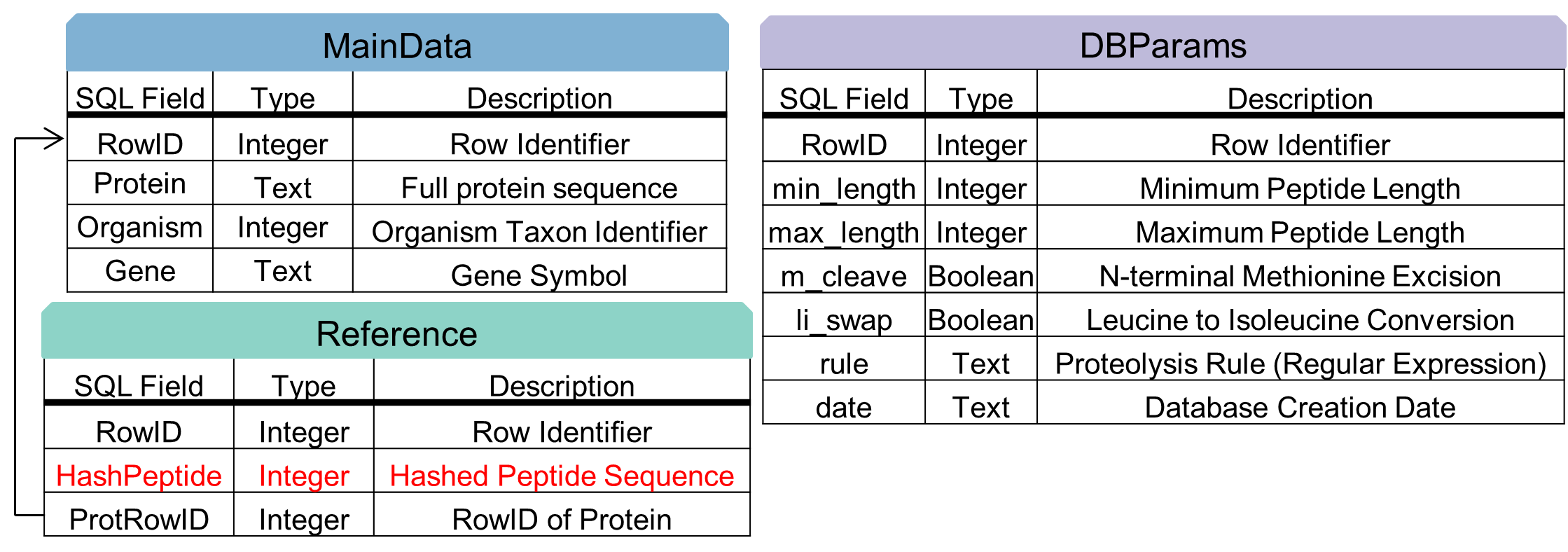

Supplement: S1 Fig — The database stores information across three tables: “MainData” stores complete protein sequences, organisms, and genes; “Reference” contains all peptide information and a key back to the protein table (black arrow); “DBParams” stores all database parameters at the time of database creation. The indexed column, “HashPeptide,” is indicated in red. (TIF) [file pcbi.1007741.s001.tif]

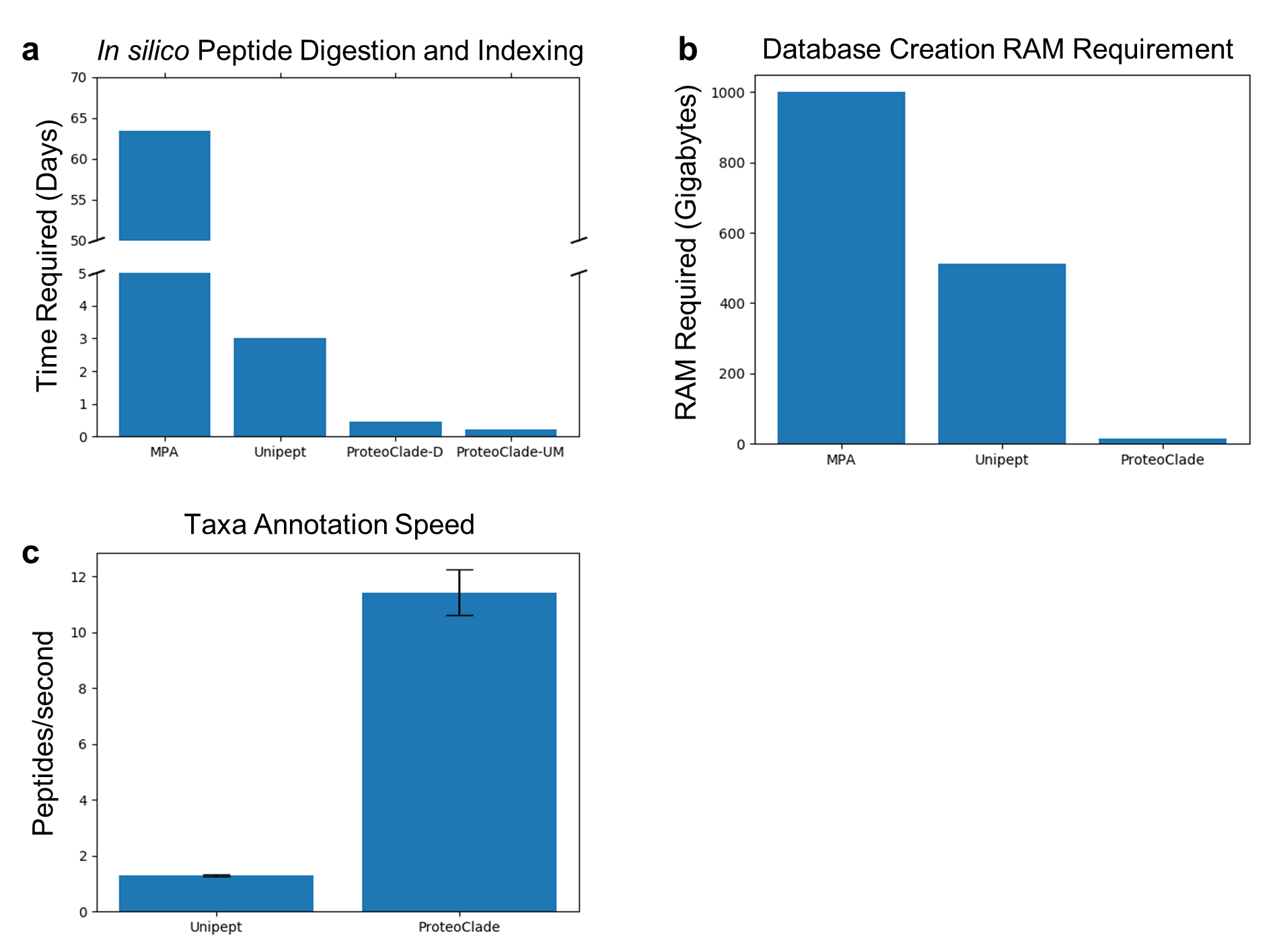

Supplement: S2 Fig — a) In silico digestion of peptides and indexing for ProteoClade’s default settings (ProteoClade-D) is faster than previous tools when using the entire UniProt repository. A database using the same parameters as Unipept (ProteoClade-UM) was faster still, due to the absence of missed cleaved peptides. b) Database RAM requirements for ProteoClade enable users to generate large databases without using high performance computers. c) Annotating all taxa for experimental results is 8.8x faster for ProteoClade than Unipept. (TIF) [file pcbi.1007741.s002.tif]
